# Selections and Abstracts

**Published:** 1901-06

**Authors:** 


### SELECTIONS AND ABSTRACTS.

### The Hospitals of Japan.*

Japan has few hospitals, only ten. This is certainly a very small number when we consider that the country has a population of forty-five million and several large cities, one as large as Philadelphia, and three with five hundred thousand inhabitants each. It has a few cities with a hundred thousand people and no hospital at all. Tokyo, the capital of the nation, only has two, the Imperial University Hospital and the General Hospital.

The former is the largest and in many respects as good as any institution of the kind I have ever seen. It is as large as all the other hospitals of Japan put together. It is almost entirely maintained by the government. It has eighty resident physicians and six hundred trained nurses. The average number of patients treated there is twenty-two hundred, and in the various out-door departments many thousand sick people are treated annually. The main building makes no pretensions to architectural beauty; it is a perfectly plain two-story brick and stone structure, a hundred feet wide and four hundred feet long. It is located in the middle of a beautiful park, with its lawns, green terraces, tropical trees and plants, playing fountains, and here and there artistically arranged and various shaped are comfortable looking rests or seats, some in the sun, others in the shade, many grouped around fountains, while some are scattered along little rippling streams. Here landscape gardening has reached the highest state of development.

This building is only used for offices, reception rooms, parlors, library, museum, billiard rooms, drug rooms, and the microscopical department. This microscopical laboratory is the largest and most complete I have ever seen. Here I had the pleasure of

*By Edw'd C. Register, M.D., President of the Board of Medical Examiners State of North Carolina, Ex-President of the Charlotte Medical Society, in *Charlotte Medical Journal.*

meeting the celebrated Dr. Kitasato who was sent, several years ago, to China and India by the Japanese government to investigate the bubonic plague, and who successfully isolated the bacillus of this disease. He is evidently a very scienttfic man and an accomplished physician.

With the exception of the operating rooms, all the other buildings connected with this institution are one-story high, made of wood, and join the rear of the large stone building, leading off from it at right angles and parallel with each other. They are four in number and extend back possibly five hundred feet. About every hundred feet they are connected with each other by covered bridges with glass sides. On both sides of all the wooden buildings, there is a narrow veranda which is usually closed by sliding glass doors. All the wooden buildings are painted white, inside as well as outside.

The physicians and nurses wear white uniforms, European in style. With all this perfectly clean and glittering glass, surrounded by so many flowers and shades, with the sun's rays peeping in here and there, it certainly looks beautiful and healthy.

Connected with these buildings, there is one for the physicians, one for the nurses, and one for the servants, a department for lying-in patients, one for contagious diseases, and one for the insane. The architecture of them all is uniform; the distance between them and the way they are connected are all identically alike. Several other buildings, used for minor purposes, are scattered about over the park, making a perfect network of houses, all conveniently arranged and magnificently kept.

The surgical department is a large two-story stone structure, plain, but rather handsome. It stands off to itself. It is a comparatively new building, has only been finished about a year. It has several operating rooms and amphitheaters, and can take care of about two hundred surgical cases at a time. Minor cases are usually cared for in the main hospital building.

Surgeons in this country are very conservative, a great deal more so than in America. Patients are slow to consent to be operated on. They have to know that it is their last chance before they will consent. This is not because they are cowards or not as brave as other people are. It is because they have acquired, and to some extent inherited, a prejudice against surgery. This is not peculiar to the Japanese, it is characteristic of all oriental semi-civilized people where Buddhism exist. Some of its former teachings prejudiced the people against surgical operations. To cause bloodshed except when favored by their god of war was a great wrong. There was no exception to this rule, even in their relations to the lower animals. To a great extent this prejudice is gradually being overcome.

This makes the surgical work of this great hospital rather small when compared with its other departments. It has septic as well as aseptic operating rooms. In the former, they pay very little attention to cleanliness, but in the aseptic operating rooms everything glitters and is in perfect order and is no doubt thoroughly aseptic. In and around these operating rooms you can see large and beautifully arranged instrument cabinets filled with every apparatus and appliance known in connection with modern surgery. The most of them are made in Japan, but they import some of them from Germany, England, and a few from America. The wards were overcrowded, and the rooms for single patients are very small, not over ten feet square, and, strange to say, in an institution so modern and so well equipped in so many respects, would furnish their first-class rooms, just as they are in a hotel, with velvet carpets, rugs, curtains, cloth-covered sofas and chairs.

The crowding of their wards to overflowing seemed to me cruel, yet the patients looked comfortable, and many of them happy. Both sexes were often in the same ward, being bathed and dressed at the same time, without any embarrassment to any one.

It has been said that nudeness can be seen in Japan more than any other place in the world, but it is never looked at. The correctness of this was impressed upon me when going through the wards of this hospital.

While the surgeons in this country are very conservative, they are not timid. Many of them do excellent work. I spent a day in this Imperial University hospital, saw several operations, and I observed nothing that was not intelligently and skillfully done. One young assistant surgeon, who could speak a little English, told me that he had used the Murphy button seventeen times without a single failure, and that the chief surgeon had performed seven laparotomies for perforation in typhoid fever and saved three cases.

I was astonished to see so many cases of tuberculosis in this hospital. Forty per cent. of the inmates had tuberculosis. Going back over the records for five years shows that thirty-five per cent. of all cases admitted were tuberculous. This great susceptibility to tuberculosis on the part of the Japanese was something new to me. Statistics show that thirty-two per cent. of all deaths in Japan is due to tuberculosis. In America it is less than fifteen per cent. and we are justly alarmed.

Rheumatism was the next most prevalent disease I found in this hospital, and skin diseases were very rare.

It is easy to observe the causes of consumption in this country. Leaving out all hereditary tendencies, the habits and customs of the people would naturally cause it to develop. Their houses are always built on the ground, uniformly one-story high, few windows and they are like pigeon-holes. They have few facilities for heating their houses. Even in the coldest weather they will do without fire, consequently their homes are cold, damp and dark, just the conditions and surroundings to favor the development of tuberculosis. Besides a Japanese seldom has anything on his floor. Sometimes among the better classes they will use a straw matting, something like we use in the summer. They always take off their sandals or wooden shoes at the door and wear nothing on their feet while in the house, no matter how cold and damp it is. With these conditions and methods of living it is not surprising that consumption and rheumatism are so prevalent.

The absence of skin diseases among the Japanese is evidently due to their cleanliness. I suppose they bathe more than any people in the world. There are over eleven hundred public baths in Tokyo alone, and it is estimated that four hundred thousand people patronize these baths daily. They use the water a great deal hotter than we do in America, seldom under 110 deg. Fah. and often 118 or 120 deg. Fah., and remain in the bath for hours, especially in the winter, as it is a cheap way to keep warm. It costs them one sen for each bath, about a half cent in American money.

I noticed in the Imperial University hospital that they were giving creosote in pulmonary tuberculosis, in seventy-five-drop doses, three times a day, injecting serums made in Germany, and experimenting with some made by themselves. They were using inhalers and sprays just as we do, and I suppose with about the same success.

The general hospital at Tokyo is quite a nice institution. It is partly under the control of the Red Cross Society of Japan. It has twenty resident physicians and two hundred trained nurses. Its average attendance is seven hundred, besides thousands of sick people are treated in its various out-door departments. The buildings are old and the grounds have an appearance of dampness and neglect, a lack of brightness that does not very favorably impress a visitor. The general arrangements of the buildings are on the cottage plan, with one very large brick building which is used for the officials of the hospital. The operating rooms are fairly well arranged and equipped. They will compare very favorably with some of our large hospitals.

The Yokohama hospital is small and badly arranged, and evidently poorly managed. It is attended by a staff of three physicians, who live in the city. The building is old, damp and dark, surrounded by no gardens or yard.

Kioto, the old capital of Japan, a city of six hundred thousand population, only has one good hospital. This is the Kioto Hospital Medical School. It is a hospital and medical college combined. They are under one management and the buildings are connected. The grounds cover ten acres and are beautiful. The buildings cover about three acres and all but one of them are made of wood, and are two-stories high. The main building is three-stories high, built of stone, and it is new and a handsome structure. Twenty-eight physicians are connected with this school and hospital. Twenty one students were graduated last March. All the physicians live in little cottages on the hospital grounds, and the students room in the main building. Three physicians from Germany and one from Holland teach in the medical department. It is partly supported by the city government.

About five years ago all of its buildings were destroyed by fire and they have only in the last year finished rebuilding them, consequently everything is new and up-to-date. They have two operating rooms not connected with amphitheater halls. I have never seen anywhere two operating rooms more conveniently arranged or more thoroughly equipped. Here pharmacy is taught as well as medicine.

Several years ago the medical school was divided into a medical school proper and a preparatory medical school. When a student begins with the preparatory studies it takes him twelve years to graduate. This hospital has the most complete hydrotherapeutic establishment of any in Japan. It occupies the basement of the main building and is thoroughly modern in every respect. It comprises a Turkish bath, vapor bath, Charcot's douche, electric baths, sulphur baths, iron baths and a suite of hot and cold baths with sprays. Annexed to this department is a completely fitted medical gymnasium.

The Doshesha Hospital at Kioto is kept up by a Canadian mission. It has no resident physician and only one trained nurse, who is from New York. Three physicians attend the hospital, each a week at a time, in rotation. They have six or eight beds fixed up especially for foreigners, and many Europeans and American have been cared for there.

Nagoya, a city of two hundred thousand population, has only one small hospital. It is a private institution, run by three rather bright, enterprising young Japanese physicians. The buildings were not originally constructed for the purpose for which they are now used. The grounds are small, no lawns, and few shades. The surroundings had a dilapidated, neglected look, and the inside was dark, damp and had a mouldy smell. Their little operating room looked neat, but was poorly furnished. They had sixteen patients, but none of them were surgical cases.

Osaka has a city hospital. I did not have an opportunity to visit it.

Kobe and Nagasaki each has a hospital. The one at Kobe interested me greatly. Its buildings are very large and it is evidently well patronized. They have eighty trained nurses, and an average of two hundred and fifty patients. Its reception rooms for out-door patients were crowded to overflowing. The general operating room for third-class patients interested me more than anything surgical I have seen in Japan. Here seven operations in one room were being performed at one time. It reminded me of Barnum's circus, it had so many attractions going on at one time. It had no preparatory ante-room for undressing or dressing. The anesthetic was administered and, in fact, everything connected with each case was done in this one room. Female as well as male patients were admitted and treated or operated on as their time came. I noticed one surgeon was operating for urethral stricture in the male, another setting a broken arm for a little boy, while another was doing gynecological work. Only seven physicians remain in the hospital at night; all the others live in different parts of the city. I could not learn how many were connected with it or how they were appointed.

The Red Cross Society has recently established a hospital in Kobe. The day I visited it, it only had three patients, one nurse, and no resident physician. I did not see the hospital at Nagasaki. I understand it is used partly for the Japanese navy. America, England and Germany all have naval hospitals at Yokohama.

I suppose it might be said that there are a great many other hospitals in Japan that I have not mentioned. There are many little mission hospitals where they are doing dispensary work, and often they have a few beds where they take care of three or four patients at a time. A great many physicians have their own little private hospitals. I visited several of them. They are so small, have so few facilities, and are so poorly patronized, that they are not recognized by the local city directories. The Japanese army has several hospitals. I did not, of course, visit them.

The Imperial Hospital at Tokyo, that I described at first, seems to be the medical center of Japan. Nearly all of the best people, throughout the country, when they have to submit to any important surgical operation, or have any serious complicated disease, go there. The distance from any part of Japan to Tokyo is short, the railroad facilities are good and the fare is less than a cent a mile. This makes the surgeons, physicians and specialists there very accessible, and they are patronized more than they are in any other part of the country.

The Japanese physician is peculiarly fitted for certain departments of medicine. It is characteristic of the best element of the race to be industrious, deliberate, careful, and he loves more than anything else to work for days, weeks, and even months, at a single little thing. Mr. East certainly knew the people well when he tersely said that they seemed to be "great in small things and small in great things."

I notice that they are enthusiastic workers in microscopy, their patience seems never to tire; they will prepare slide after slide, specimen after specimen, and their interest never sags. This kind of work suits them.

In surgery the smaller and more delicate and difficult the operation is, the more it interests them. The average Japanese physician would rather see a cataract operation than a hysterectomy. To watch them prepare for an operation, the time they seemingly throw away arranging little things, the minute instructions they give their assistants and nurses, even in minor surgical cases, and to observe them fix, with so much care and deliberation, every table and tray, every knife and sponge, perfectly oblivious to time, is as amusing as it is tiresome to the hustling, restless, and impatient American.

Nagasaki, Japan, Oct. 23, 1900.

---

### CLINICAL EXPERIENCE WITH ADRENALIN.

BY EMIL MAYER, M.D.

Surgeon New York Eye and Ear Infirmary, Throat Department; Fellow American Laryngological Association; and of the New York Academy of Medicine, New York. Abstract from original paper, in the *Philadelphia Medical Journal*, April 27, 1901.

The aqueous extract of suprarenal gland is perhaps the best culture medium known. Its instability, the involved method of preparation, its unsightliness, and the inexactitude of its various strengths tend to make us welcome a preparation that is exact, stable, and above all, clean. Dr. Jokichi Takamine undertook the task of isolating the active principle of the suprarenal gland. He obtained a substance in stable and pure crystalline form, which raises the blood pressure, and which he named "Adrenalin."

The author has used solutions of Adrenalin Chloride, 1 to 1,000, 1 to 5,000, and 1 to 10,000; his cases were all rhinological. Blanching of tissues followed the application of the strongest of these solutions in a few seconds, and was very thorough. In no instance was there any constitutional disturbance. He has employed no suprarenal extract since for any purpose whatever.

The effect of the solutions was not altered by their change to a pink color; they were used for six weeks. Subsequently a small amount of chloretone was added to the fresh solutions, and now there is but slight change of color and no floccules appear.

Thirty-five cases are reported in tabulated form, showing that the usual effect of the aqueous extract of the suprarenal gland was obtained. A few operative cases bled freely, but in every instance the hemorrhage was promptly checked by a second application of Adrenalin. The Adrenalin was used not only as a hemostatic, but for the relief of nasal congestion, as a diagnostic aid, and for the continuous treatment of acute inflammatory affections of the accessory sinuses.

The author arrives at the following conclusions:

1. Adrenalin Solutions supply every indication for which the aqueous extract has been used.
2. They are sterile.
3. They keep indefinitely.
4. Solutions, 1 : 1,000 are strong enough for operative work; and 1 : 5,000 and 1 : 10,000 for local medication.
5. They may be used with safety.

In this connection it is interesting to note that E. Fletcher Ingals, M.D., of Chicago, also has had a very satisfactory experience with Adrenalin. In a paper entitled, "Notes on Adrenalin and Adrenalin Chloride,"* he reports that he experimented with solutions, varying from 1 to 1,000 to 1 to 10,000, of the Chloride of Adrenalin in distilled water or normal salt solution, and kept careful records until satisfied of its activity. In nine cases a very

*Journal of the American Medical Association, April 27, 1901.

small quantity of a spray, of one part of Chloride of Adrenalin to 10,000 parts of water, was applied to the nasal cavities, with the effect of blanching the mucous membrane quickly, and in most cases causing contraction of the swollen tissues similar to that caused by cocain. The first solution used was made with distilled water and caused smarting; normal salt solution was then used as the solvent with perfect satisfaction. The smarting may have been due to the presence of a small quantity of formalin in which the atomizer had been washed just before use.

Experiments were also made with insufflations of a dry powder consisting of 1.5 per cent. (75 parts) each of biborate of sodium and bicarbonate of sodium; 3 per cent. (150 parts) light carbonate of magnesium; one part of Adrenalin to 5,000 parts sugar of milk. This powder cleared the nasal cavities when obstructed by swelling of the turbinated bodies, and diminished the secretions decidedly. A case of daily epistaxis was relieved by sprays of a 1 to 10,000 solution. Another of conjunctival congestion from overwork was entirely relieved by the instillation of a similar solution. The author has had equally satisfactory results in cases of conjunctivitis; laryngitis, acute and chronic; acute laryngitis with edema glottidis; acute coryza; chronic laryngo-tracheitis with acute exacerbation ; and in preparation for operations upon the nose.

In conclusion, the following results are presented : This remedy will be of great value in the treatment of acute inflammatory affections of the nasal cavities, either in sprays of 1 to 5,000, or in powders of 1 to 5,000, or 1 to 2,500 sugar of milk. In acute coryza and in hay fever, in epistaxis from various causes, in acute inflammation of the fauces, solutions of 1 to 1,000 will have good effects. In acute or subacute laryngitis, solutions of 1 to 1,000 applied with moderate force will give very great relief. It appears probable that vocalists may obtain sufficient relief from congested cords for at least two or three hours, to obtain normal efficiency in the use of the voice.

In a paper read before the Chicago Laryngological and Climatological Association, W. E. Casselberry, M.D., called attention to the fact that Adrenalin Chloride Solution is clear, colorless, odorless, sterile and stable if protected from heat, light and oxidation ; it is non-irritating to mucous membranes. When applied locally it exerts identically the same vaso-constrictor influence as the aqueous adrenal extract. Sprayed into the nostrils in the strength of 1 to 10,000 it produces a visible change from turgidity to compactness of the turbinated tissues, and a decided pallor of the mucous surfaces. In the strength of 1 to 1,000, or even 1 to 5,000, it has the power to limit hemorrhage during operations and is an aid in the treatment of epistaxis. It may be substituted for cocain in all cases in which an ischemic effect is desired—*e. g.*, to facilitate inspection of the deeper recesses of the nasal cavities and to make them more accessible. Adrenalin has little or no cerebral stimulant effect, exciting no desire for more of the drug; hence there is little risk of habit-formation.

The author expresses the opinion that Adrenalin should afford relief in asthma associated with bronchitis and vaso-motor paralysis, although he would expect little benefit from its use in asthma characterized by bronchial spasm. It may be formed into an ointment with vaseline, or mixed with stearate of zinc, powdered starch, or sugar of milk to make powders for nasal or laryngeal insufflation. The bibliography is very comprehensive, covering the literature of the subject down to the present date.

---

### THE PRACTICE OF MEDICINE AS A SOURCE OF INCOME.*

Hipprocates says: "Godlike is the physician who is a philosopher;" and Aristotle says: "The philosopher should end with medicine, the physician begins with philosophy;" and when I consider my text, "The Practice of Medicine as a Source of Income," I suppose I am expected to philosophize on how to get a living out of the practice of medicine—sometimes a rather difficult proposition, a proposition that to many of my audience I have no doubt has given rise to the most serious study and contemplation, not merely from the standpoint of gaining wealth, fame, and position, all supremely desirable attainments in the practice of medicine, but also sometimes from the view-point of how to make ends

*Read before the Physicians' Club of Chicago, January, 1901, by D. A. K. Steele, M.D., Chicago, Professor Principles and Practice of Surgery and Clinical Surgery, College of Physicians and Surgeons, Chicago (University of Illinois).

meet: how to meet the wants of a growing family. For while it is true that many of us start out imbued with the idea that we are members of a learned profession, and feel chock-full of the science and art of curing disease, and are only anxious for an opportunity to alleviate the aches and pains of suffering humanity, we soon learn that there is a business side to the practice of medicine, and that we must live by, as well as for, our profession. While the altruistic side of our profession has been constantly presented to us during our pupilage, we soon learn that there is a commercial side to it as well, and that in the twentieth century physicians sell their knowledge to their patients at so much a visit or so much a consultation—the *quid pro quo* being commensurate with the services rendered. Our calling has its origin in human suffering. If there was no disease there would be no physicians. As Bishop Spaulding has said, "It is our unenviable lot to live like moral cannibals on the misfortunes and weaknesses of our fellow men." But in this position we have also the company of the lawyers and ministers, who, equally with ourselves, enjoy a similar cannibalistic feast off their fellow men's moral, mental, or physical infirmities; and we also soon learn that gratitude for our services is short-lived, for Byron says: "Physicians mend or end us *secundum artem*, but although we sneer in health, when sick we call them to attend us without the least propensity to jeer." And another couplet reminds us "That when the devil was sick a saint was he, but when the devil was well, devil the saint was he."

It is with regret that I note a tendency in modern civilization, in the fierce struggle for existence on the part of the medical profession, to relax those rigid rules of regard for medical ethics and adherence to the golden rule that, during the early part of the last century, was a marked characteristic of our profession. I fear that the modern commercial spirit is driving out of our hearts and minds those high ideals of a physician that are so well portrayed in Dr. Roger Chillingworth, a character in Hawthorne's Scarlet Letter. He says: "Made up of earnest, studious, thoughtful, quiet years bestowed faithfully for increase of knowledge, faithfully too, for the advancement of human welfare. Men thoughtful for others, caring little for themselves; kind, just, true, and of constant, if not warm affections." Such a faithful picture of the early New England physician; quite different from the modern city doctor, the Chicago type, who already shows in a marked degree the degrading influence of modern commercial methods of dishonest commissions; of questionable newspaper interviews; of countless reprints for the allurement of prospective patients; and all the other things that specialists especially have done to degrade our profession and enhance their own income.

Division of labor makes everything cheap—man first of all; and the increase of specialists has had the effect to lower the standard of professional life and morals, and to interfere with the development in the profession of strong, many-sided personalities, lending dignity to their calling, and yet masters in their several departments. As Henry George says, "I am for men.'' My ideal is a doctor who is educated all over, whose heart is tender, whose hand is steady, whose eye is clear, whose tongue is clean, whose brain is cultured, whose nerves are under perfect control—one who is broad-minded, and who does not look at disease through the narrow view of the specialist, who sees only from his own field of vision. I want a doctor whose knowledge of disease has been broadened and deepened and enriched by a wide and varied experience in general medicine, and sharpened and polished in some specialty afterwards—a doctor who has a had a hospital experience, who mixes common sense with knowledge and experience, who has a heart swelling with sympathy for the poor sufferers who seek his aid, who carries a smiling face on his errands of mercy, who prefers substance to show, and who regards reputation as a priceless treasure, to be guarded against the alluring temptations of modern society, or the tempting bait of gold offered for the prostitution of his talents to the performance of illicit or illegal practices. I demand that he shall possess that innate monitor that we call conscience. And while it is true that there are many in our profession who accumulate wealth, who do not possess the characteristics I have portrayed, we must remember that there is much more in our lives than the mere acquisition of money.

In regard to "the practice of medicine as a source of income," there is a vast amount of ignorance on the part of the laity, and of the profession as well, that is usually rudely dispelled when the dead doctor's will is made public in the probate court. And general surprise is expressed that Dr. A——, who was reputed to have an income of from $20,000 to $30,000 a year, only left his family a $10,000 estate, and had allowed his life insurance to lapse a few months before his death; and that Dr. B——, who was a celebrated specialist, had left practically nothing to his family; and that Dr. C——, one of the best known general practitioners in the city, left only debts as a reminder of his activities.

I have taken some pains to ascertain the average incomes of doctors from their practice, and am of the firm conviction that professional incomes are greatly overestimated. And it is a wise physician who provides for the future needs of his family by carrying a liberal amount of reliable life insurance, as very often that is the only estate he leaves them, for his expenses fully keep pace with his increasing business—larger incomes demand better offices, a finer house, more servants, more social duties, and at the end of the year the balance-sheet shows very little on the side of net profit. The $2 to $3 per visit, or office consultation, $5 to $25 per consultation, $10 to $30 for obstetrics, and the larger fees provided for operative work in the "fee table," do not insure large incomes for many in the profession. The first five or ten years' practice is usually a struggle for existence, the second ten years a competency, or good living, and the third ten years we are likely to be crowded out by the younger and more progressive element.

As a source of income, the practice of medicine offers much work, great anxiety, and moderate returns. Twenty-five per cent. of medical graduates abandon the profession for other fields of labor within five years; twenty-five per cent. exist by the practice of the profession; forty per cent. make a comfortable living, and are in easy circumstances during their professional activity, but do not leave their families well provided for when death overtakes them; seven per cent. accumulate a competency and enjoy a large income; while only about three per cent. succeed in reaching very large incomes. You can count on the fingers of one hand the practitioners in Chicago whose incomes from practice exceed $30,000 per annum, and probably not more than a score exceed $20,000 a year.

Take the average general practitioner, and the incomes vary from $1,500 to $3,000. Office specialists—eye and ear, nose and throat—average $2,000 to $6,000; consulting physicians, $5,000 to $15,-000; not more than six leading physicians, $15,000 to $35,000; not more than six leading surgeons, $20,000 to $60,000; not more than six leading gynecologists, $10,000 to $20,000; not more than six leading office specialists, $10,000 to $15,000; ten average surgeons, $3,000 to $10,000.

One's income may be enhanced in a variety of ways that are perfectly legitimate: such, for example, as increasing reputation by original work, by essays and discussions in medical societies, by affiliation with medical colleges in a teaching capacity, by becoming attached to the staff of a reputable hospital, by caring for the sick or injured of a corporation, by making life insurance examinations, etc. And it may be increased illegitimately by criminal curettements, by unnecessary operating, by useless visits, by exploiting cases in the public newspapers, by specialists' reprints for patients, by lending one's name to indorsement of proprietary remedies, etc. The old saying that "honesty is the best policy, but it keeps a man mighty poor," is exemplified in the medical profession, where we are constantly practicing preventive medicine, preventing epidemics, destroying the causes of disease, teaching the public the limitations of our art; and our honesty is rewarded by less disease, less sickness, a lowered death-rate, and the growth of numerous fads, such as Christian Science, Dowieism, faith-cures, etc.

The laborer is worthy of his hire, and we owe a duty to ourselves, to our families, and to society, to put our services upon a business as well as a charitable basis. The public will place much the same value upon your services that you do yourself. If you are careless or indifferent about collecting, your patrons will be careless and indifferent about paying.

Always have a straightforward and accurate understanding with your patients as to the value you place upon your services. Never tell a patient when he offers to pay a bill, "Oh! never mind, to-morrow will do just as well." Now is the accepted time; to-morrow may never come. Gratitude is short-lived. Our charges for professional services should be somewhat proportionate to the ability of the patient to pay, and to the responsibility involved in the services rendered in a given case. Keep a neat, legible set of books; keep your accounts posted up each week; present your bills monthly, and if they are not paid promptly, give them to an honest collector. Keep everything up in a businesslike way, and your patrons will esteem you more highly. By being businesslike in your dealings with patients you will make your profession honored; you will make the practice of medicine a source of income; and you will derive the satisfaction, not only of the scientific pursuit of knowledge in the practice of our art, the keen enjoyment of original research, but the added satisfaction of deriving a living income from your efforts.—*Medical Age.*

---

### CHRISTIAN SCIENCE AND CONSUMPTION: CAN THE LATTER BE CURED BY THE FORMER?

During recent litigation over the will of the late Miss Brush, member and healer of the First Church of Christ, Scientist, attention seems to have centered on the question, "Was Miss Brush at the time of deeding $65,000 to Mrs. Stetson's church of sound mind?" I will leave the decision to my confrères, the specialists and experts in diseases of the mind.

To me, an ordinary physician, interested in more common diseases, and perhaps a little more so in consumption, because it is the most common, a condition of affairs was revealed during the hearing of the testimony of Mrs. Augusta E. Stetson, "Chief Healer, first reader and business manager of the First Church of Christ, Scientist," which truly alarmed me, and must alarm every physician, sanitarian, statesman and student of the tuberculosis problem.

Of the many remarkable statements made by Mrs. Stetson in Surrogate Fitzgerald's court, I will quote as an introduction to this appeal to the intelligent men and women of this country only the following:

"That book can, I believe, cure anybody who reads it understandingly of any disease, even of consumption."

"Miss Brush had consumption when I called on her in June.

At her request I directed Dr. Pease, who was in charge, to leave, and took myself charge of the patient. She died in July."

The book referred to was Mrs. Mary Baker Eddy's "Science and Health," and the Dr. Pease mentioned was Charles G. Pease, M.D., D.D.S., a gentleman practicing dentistry, but also a convert to Christian Science, and who helped to overcome a legal difficulty by signing the death certificate.

Now, let us ask what, in the light of reason and modern research, such practice as is exemplified by Mrs. Stetson's testimony really means.

Consumption, or pulmonary tuberculosis, is a chronic and highly communicable disease. The medium of contagion is the tubercle bacillus. This micro-organism is ejected by the coughing consumptive with the expectoration, and if no hygienic care is taken, if this patient expectorates indiscriminately, the infected sputum when dried and pulverized may be inhaled with the dust of the atmosphere and pulmonary or laryngeal tuberculosis may thus be contracted. A consumptive, up and about, even if in the earlier stages of the disease, may expectorate as many as seven billions bacilli in twenty-four hours.

It is now universally understood by the greatest authorities of Europe and the United States that the careless and indiscriminate manner in which consumptives so often dispose of their expectoration is the principal cause of the propagation of the disease. According to Professor Osler, of Johns Hopkins University, there are over 1,250,000 cases of consumption in the United States all the time, of which I venture to say, at least thirty to forty thousand are in the city of New York alone. The death-rate from this disease is correspondingly high; in some States every sixth, in others every seventh, death is due to pulmonary tuberculosis.

Since 1882, when Professor Robert Koch, of Berlin, discovered the bacillus of tuberculosis, thus making the recognition of the disease more certain, the number of deaths from tuberculosis on this continent alone has been estimated to have been no less than two millions.

Thanks to the discoveries of modern medical science, we are, however, no longer powerless against this scourge of the human race. Consumption is no longer considered a divine visitation to which we must meekly submit with folded arms. The researches in the laboratory and the experiments with the open air treatment have shown that tuberculosis in many of its forms, but particularly in the form known as consumption or pulmonary tuberculosis, is a decidedly preventable and in many instances very curable disease.

The modern treatment of consumption consists in placing the patient in the very best hygienic conditions possible, in either the private home or the sanitarium. He will have to live virtually all day and all night in the open air. The very best of food and constant supervision on the part of the medical attendant will do the rest to reestablish the health of the patient.

Now let us study for a moment the book which, according to Mrs. Stetson, can, when read understandingly, cure consumption. In chapter 2, on page 58, of "Science and Health," latest edition, we read: "Obedience to the so-called physical laws of health has not checked sickness. Diseases have multiplied since man-made theories have taken the place of primitive Truth."

We can easily refute the statement that obedience to the so-called physical laws of health has not checked sickness. Since we are dealing with tuberculosis in this article, I will confine my statistics to this disease. In England for over fifty years excellent hygienic laws have existed, and for an equal number of years there have been special institutions for the treatment of consumptives. The following is the result of England's work in the combat with tuberculosis:

The death-rate per million of the population of England and Wales from pulmonary tuberculosis:

| | |
|---|---|
| In 1870 was | 2,410 |
| In 1875 was | 2,202 |
| In 1880 was | 1,869 |
| In 1885 was | 1,770 |
| In 1890 was | 1,682 |
| In 1893 was | 1,468 |
| In 1894 was | 1,385 |
| In 1895 was | 1,398 |
| In 1896 was | 1,307 |

In France, Austria and Russia, where the general hygienic laws until recent years were few or none, and where special institutions for the treatment of consumptives did not exist, the mortality from tuberculosis has been on the increase.

Diseases have not multiplied in reality. A better knowledge of pathology has taught us to differentiate more understandingly. What was formerly thought to be simply inflammation of the bowels comprises to-day peritonitis, enteritis, appendicitis, etc.

On page 116, Mrs. Eddy says: "We hear it said, 'I exercise daily in the open air, I take cold baths in order to overcome a predisposition to take cold, and yet I have continual colds, catarrh and cough.' Such admissions ought to open people's eyes to the inefficiency of hygiene and induce them to look in other directions for cause and cure."

A more erroneous statement can hardly be conceived. Because some one had probably overdone the exercise in the open air, and taken his cold douches too cold and without receiving the valuable benefit of this excellent hygienic means of preserving health and even preventing such diseases of consumption, Mrs. Eddy, Mrs. Stetson, and their followers declare themselves against the use of God's fresh air and cold water as a means to remain or get well. When the educated physician recommends cold baths as a means of overcoming a disposition to take cold, he will tell his patient at what temperature he should commence these hydrotherapeutic applications, how gradually to decrease the temperature and what precautions to take in order to assure proper reaction.

I would not expect either Mrs. Eddy or Mrs. Stetson to have even the faintest understanding of hydrotherapy, a branch of medicine which must be studied as well as any other.

We have said that the best of food should be given to the consumptive patient; but not only to give the best, but an abundance—well-nigh an overabundance—is an essential part of the modern treatment of consumption. The tendency to waste of tissue, so characteristic of all consumptives, can only be overcome by judicious overfeeding. And what does "Science and Health" say about food? On page 387 we read: "The fact is, food does not affect the real existence of man."

Every physician who has treated consumptives will bear me out when I say: The fact is that food does affect the real existence of the consumptive patient more than anything else, and that without an abundance of good food the consumptive will never get well.

Among the earlier signs of consumption we not infrequently note an expectoration of a blood-stained mucus, or even a real hemorrhage from the lungs. To thousands of consumptives these early hemorrhages have been the salvation. They became alarmed, consulted their physician, and by immediate, judicious and rational treatment their lives were saved. What does Mrs. Eddy counsel the young woman "inspecting the hue of her blood on a cambric handkerchief" (page 378)? After recalling to her the experiment of English students with a felon who fancied himself bleeding to death and died, she tells this consumptive patient to "learn the opposite principle of life as taught in Christian Science," that "she is but suffering from her belief that blood is destroying her life, the so-called vital current does not affect the invalid's health, but her belief produces the very results she dreads." In other words, instead of seeking means to stop the flow of blood and by judicious and timely treatment prevent a recurrence, Mrs. Eddy tells the consumptive sufferer no matter how much blood she loses her health cannot become affected thereby.

But the most remarkable and dangerous of all the teachings of Christian Science is the one found on page 388 of "Science and Health," which reads: "The less we know or think about hygiene, the less we are predisposed to sickness." In this teaching lies the greatest danger which comes to us from Christian Science. To allow men and women, after taking a six weeks' course in Christian Science, using as a text-book the volume from which I have quoted, to be the sole attendant of persons suffering from a disease as highly communicable as pulmonary tuberculosis seems to me absolutely dangerous.

No matter, how great their ordinary intelligence may be, the moment they accept as a guide in the treatment and prevention of diseases the tenets that are set forth in the book "Science and Health," they are not only utterly unable to treat a consumptive, or for that matter any other patient, successfully, but by their disregard of hygienic laws they help the propagation of tuberculosis and other contagious diseases. They thus commit a crime against their fellow men and render futile all the work of the sanitary authorities who are trying their very best to stamp out such diseases.

It has been claimed by Christian Scientists that those opposing their creed and practice forget that Christian Science is a religion, and that the followers of this religion have a right to exercise the rights appertaining to that sect. Far be it from me to speak one unkind word against the purely religious aspect of the doctrine of Christian Scientists. They have a right to trust in God, and with whatever their simple belief and childlike confidence may add to their individual happiness we have no right to interfere.

But we cannot allow them, either by example or practice, to trifle with the nation's health. Every intelligent man and woman must realize that such doctrines as an utter disregard for hygiene and prevention of contagious diseases must become a peril to a nation.

Christian Scientists claim not to practice medicine because they do not give medicine; but by not giving medicine when medicine should be given, by allowing diseases of a contagious nature to propagate through either carelessness, ignorance, or disregard, they commit malpractice, and thus violate the law. Christian Science as a religion may be beautiful and comforting to some; as a religious sect treating diseases with which they may be afflicted themselves, or from which their children and friends may suffer, they preach and practice a pernicious doctrine. No physician underestimates the value of mind over a mind diseased or nervous diseases of an ordinary character, but it requires years of training and study to be able to distinguish such from true organic affections. Christian Scientists do not make any distinction between imaginary, functional or organic diseases. They treat all alike.

If Christian Scientists are really in earnest in their declaration that their only desire is to uplift humanity, let them direct their uplifting influence toward overcoming the drink habit, the gambling habit, the criminal tendency among old and young, and other vices. Let them cure, by present or absent treatment, the various social ills, such as indifference to the sufferings of our fellow men, avarice, vulgarity, etc. Let them come forward with their wealth, and instead of building marble churches, let them build model tenement houses, so that their brothers and sisters may live like human beings.—*S. A. Knopf, M.D., New York, in Journal of Medicine and Science.*

### HYPERACIDITY A CAUSE OF SKIN DISEASES.

In a paper entitled as above, presented to the Section on Cutaneous Medicine and Surgery, at the fifty-first annual meeting of the American Medical Association (*Journal of the American Medical Association,* March 30, 1901), W. R. Inge Dalton, M.D., of New York City, says, in part:

"There are other etiologic factors, such as the physiological process of reflex irritation, of dentition, hard fecal accumulations, pregnancy, epileptiform convulsions, shocks, worms, phimosis, adenoids, etc., causing, temporarily, tendencies to morbid conditions of the skin, but the *causa causarum,* the main etiologic element—I proclaim it boldly—is *hyperacidity of the chyle in the duodenum;* to this is due many ills to which the skin is heir. I have based my treatment upon this etiologic theory, and have met with phenomenal confirmation of its efficacy in my service at the Metropolitan Hospital, and in private practice, in the various forms of eczema, pemphigus, etc. I prescribe:

| | |
|---|---|
| Naphthalin | 1 grain. |
| Ipecac | 1 grain. |
| Charcoal | 1½ grains. |
| Strychnia | |
| Pilocarpine | |
| Arsenious acid | āā 1-100 grain. |

"The naphthalin causes antisepsis and inhibits the action of micro-organisms through the ileum and large intestine, thereby arresting organic fermentative changes. The charcoal, besides being a splendid antiseptic, converts the anerobic condition of putrefaction into compounds which are not destructive to the tissues; the mercury, whether it acts upon the liver or not, destroys the bacterial forms in the duodenum and jejunum and lowers the blood-pressure, thereby accelerating capillary circulation, thus favoring digestion and obviating putrefaction; the pilocarpine and ipecac exert, at the same time, their influences upon the sweat glands and lymphatics; and finally the strychnine tones up the vasomotors and the whole cutaneous nervous system. I do not claim that my remedy will kill the pathogenic microbes in every instance, but it *does* limit their morbid energies, and by so doing gains time for the conservative remedial agencies of the organism to perform their normal function. Some pathogenic bacteria, which elaborate toxins or ptomaines in septic or putrefactive conditions, are otherwise entirely innocuous. We can unquestionably lessen their virulent activities by altering the environments of the pathogenic organisms. The stomach must be in proper condition, and suitable diet enjoined; otherwise, no matter how specific the remedy may be, if the sympathetic nerve centers, governing metabolic processes, standing for assimilation, are held in check from ptomaine intoxication, no good effects can follow. Obstipation of the bowels must in every case be combated, as uric acid accumulates in the blood. Horbaczewski and Weintraud have proved that uric acid is formed wherever living matter exists, by the dissimilation of albuminoids as demonstrated by Kossel, passing through the intermediate stages of nuclein and xanthin to uric acid. I generally follow my tablets by giving sulphate of magnesia, carbonate of lithium, and phosphate of soda, in an effervescent form the next morning."—*Medical Age.*

---

### RULES FOR DYSPEPTICS.

1. Eat slowly, masticating the food very thoroughly, even more so if possible, than is required in health. The more time the food spends in the mouth, less it will spend in the stomach.

2. Avoid drinking at meals; at most, take a few sips of warm drink at the close of the meal, if the food is very dry in character.

3. In general, dyspeptic stomachs manage dry food better than that containing much fluid.

4. Eat neither very hot nor cold food. The best temperature is about that of the body. Avoid exposure to cold after eating.

5. Be careful to avoid excess in eating. Eat no more than the wants of the system require. Sometimes less than is really needed must be taken when digestion is very weak. Strength depends not on what is eaten, but on what is digested.

6. Never take violent exercise of any sort, either mental or physical, either just before or just after a meal. It is not good to sleep immediately after eating, nor within four hours of a meal.

7. Never eat more than three times a day, and make the last meal very light. For many dyspetics two meals are better than more.

8. Never eat a morsel of any sort between meals.

9. Never eat when very tired, whether exhaused from mental or physical labor.

10. Never eat when the mind is worried or the temper ruffled, if possible to avoid doing so.

11. Eat only food that is easy of digestion, avoiding complicated and indigestible dishes, and taking but one to three kinds at a meal.

12. Most persons will be benefited by the use of oatmeal, wheat meal, cracked wheat, and other whole-grain preparations, though many will find it necessary to avoid vegetables, especially when fruits are taken.—*Public Health Journal, April, 1901.*

---

### FATALITIES OF SPINAL COCAINIZATION.

P. Reclus states that spinal cocainization now has a record of six deaths in Europe. Goilav and Jonnesco, of Bucharest, have each reported a fatality. In the former's case 1.5 cg. of cocain was injected and a leg amputated. Two hours later the temperature rose to 38 and 40 C., pulse 125 and death in twenty hours. Juilliard has also reported a death the second day after an operation for hydrocele and inguinal hernia. The autopsy showed a ruptured aneurysm of the Sylvian artery. The vasoconstriction induced by the cocain may have been a factor in the premature rupture of the aneurysm. Even in Tuffier's case, in which a mitral lesion and acute edema of the lung have been assigned as the cause of death, Reclus queries whether the action of the cocain may not have been a factor in the evolution of the edema. Heumberg has also reported the death of a man of thirty in coma fifteen days after an operation under spinal cocainization. The autopsy disclosed hemorrhage in the cauda equina. In Dumont's case a febrile, tuberculous lad in bad general condition died two days after spinal cocainization, and no direct cause for the death could be discovered at the autopsy unless it were the cocain. This total of six deaths to less than 2,000 applications of spinal cocainization is not an encouraging record, he remarked, in the conclusion of his address to the Paris Académie de Médecine, March 19th.—*Ex.*

---

### THE USE OF FORCEPS IN CHILDBIRTH.

There are times when the use of the forceps is proper and most advantageous both to mother and child. There are times when their use is utterly uncalled for. In other words they are valuable implements in their place. Hugh Boyd, in the *Alabama Medical Journal*, says that Carpenter tells us they are called for: 1. "Whenever, in head presentations, normal labor becomes difficult or impossible from feebleness or absence of expulsory pains; (2) whenever there exist a disproportion between the child and maternal pelvis—as excessive size of fetal head—prematurely ossified head, contracted pelvis or excessive resistance of perineum; (3) whenever there exists any dystocis, as hemorrhage, syncope, eclampsia, rupture of uterus—in any condition in which the life of the mother or the child is endangered by its remaining; (4) sometimes in breech presentations. This eminent authority states further that they are called for more frequently in cases of (1) resistant perineum, (2) persistent occipito-posterior positions, and (3) contracted pelvis, than in any others; and of these the first two are most common—especially in primipara."

---

### TOXEMIA OF PREGNANCY; ITS DIAGNOSIS AND TREATMENT.

In a long, comprehensive and able article upon the above subject, S. Marx, in the *Record*, draws the following conclusions:

1. Toxemia of pregnancy is a complex condition depending on more than one factor.

2. Many women go to term with albuminuria, without symptoms referable to a toxemia. When such symptoms arise they are not caused by the albumin present, but by faulty urea secretion.

3. In the most desperate and malignant cases there is found neither albumin nor casts.

4. Urea is always found markedly diminished in the so-called true toxemias of pregnancy, or urinemias.

5. Finally, I make a strong plea for a regular and methodical course of urea estimation in all cases of toxemia, or for the relegation to secondary importance of the time-honored examination for albumin.

6. Progressive diminution of urea excretion, with or without albuminuria, is the sole indication for the induction of premature labor, which is especially indicated when conscientious medical treatment fails.

---

### WHY DOES AN ACUTE ABSCESS GET WELL.

The possible reasons of the healing of an acute abscess induced by staphylococcus pyogenes aureus is partly given by G. L. Cheatle, in the *Indian Lancet*, in his words: "All that one can say at present is that an acute abscess gets well because the dose and virulence, with other factors connected with the micro-organism and the condition of the host, are so balanced that death of the host is not possible unless that balance is destroyed by a concomitant fresh infection or failure of the host to maintain its protecting properties."

---

### THE TREATMENT OF GONORRHEA BY HOT SALINE SOLUTION.

Dr. Woodruff, according to the *Medical Press and Circular* for April 10th, recommends the use of injections of saline solution, as hot as can be borne, every two or three hours, and even, if possible, every hour. Dr. Woodruff finds no necessity, under this treatment, for the administration of medicines, by the mouth, and states that the average duration of the disease in a number of soldiers thus treated was ten to twelve days only.

